# Protective Effects of Ethanolic Extracts from Artichoke, an Edible Herbal Medicine, against Acute Alcohol-Induced Liver Injury in Mice

**DOI:** 10.3390/nu9091000

**Published:** 2017-09-11

**Authors:** Xuchong Tang, Ruofan Wei, Aihua Deng, Tingping Lei

**Affiliations:** 1 College of Chemical Engineering, Huaqiao University, Xiamen 361021, China; rovanwei@yahoo.com; 2 College of Life and Environmental Science, Hunan University of Arts and Science, Changde 415000, China; dengaihua@yahoo.com; 3 College of Mechanical Engineering and Automation, Huaqiao University, Xiamen 361021, China; tplei@hqu.edu.cn

**Keywords:** artichoke, acute alcohol liver disease, oxidative stress, inflammation, TLR4/NF-κB pathway

## Abstract

Oxidative stress and inflammation are well-documented pathological factors in alcoholic liver disease (ALD). Artichoke (*Cynara scolymus* L.) is a healthy food and folk medicine with anti-oxidative and anti-inflammatory properties. This study aimed to evaluate the preventive effects of ethanolic extract from artichoke against acute alcohol-induced liver injury in mice. Male Institute of Cancer Research mice were treated with an ethanolic extract of artichoke (0.4, 0.8, and 1.6 g/kg body weight) by gavage once daily. Up to 40% alcohol (12 mL/kg body weight) was administered orally 1 h after artichoke treatment. All mice were fed for 10 consecutive days. Results showed that artichoke extract significantly prevented elevated levels of aspartate aminotransferase, alanine aminotransferase, triglyceride, total cholesterol, and malondialdehyde. Meanwhile, the decreased levels of superoxide dismutase and glutathione were elevated by artichoke administration. Histopathological examination showed that artichoke attenuated degeneration, inflammatory infiltration and necrosis of hepatocytes. Immunohistochemical analysis revealed that expression levels of toll-like receptor (TLR) 4 and nuclear factor-kappa B (NF-κB) in liver tissues were significantly suppressed by artichoke treatment. Results obtained demonstrated that artichoke extract exhibited significant preventive protective effect against acute alcohol-induced liver injury. This finding is mainly attributed to its ability to attenuate oxidative stress and suppress the TLR4/NF-κB inflammatory pathway. To the best of our knowledge, the underlying mechanisms of artichoke on acute ALD have been rarely reported.

## 1. Introduction

Drinking alcohol has always been deemed essential in many areas, such as in social gatherings, status functions, personal interactions, and conformance. However, long-term excessive alcohol intake can result in alcoholic liver disease (ALD). ALD is the leading cause of cirrhosis and liver-related death worldwide for decades and is responsible for 4% of global mortality [1,2,3]. ALD encompasses a histological spectrum of liver injury that ranges from steatosis (fatty liver) to alcoholic steatohepatitis (ASH), and in severe cases, fibrosis, cirrhosis, and ultimately hepatocellular carcinoma [4,5]. Thus, the control of ALD at an early stage, for example, at a stage prior to the occurrence of ASH, could be of great significance in preventing development of ALD.

Potential mechanisms of acute alcohol-induced liver injury are associated with oxidative stress, steaotosis, endotoxin, dysregulated immunity, and inflammation. However, in recent decades, studies have focused on the inflammatory pathway in ALD, and increasing evidence demonstrates that ASH is caused by the lipopolysaccharide (LPS) binding to toll-like receptor (TLR)-induced nuclear factor-kappa B (NF-κB) activation pathway [6,7,8]. TLRs are pattern-recognition receptors that enable the innate immune system to react immediately to infections by recognizing both bacterial and viral constituents. Among the TLRs, TLR4 can initiate activation of NF-κB and cascade response further causing the accumulation of pro-inflammation cytokines, ultimately resulting in the aggravation of inflammatory progress [9,10,11,12].

Although various treatments, such as nutritional therapy, pharmacological therapy, psychotherapy, and surgery, are currently available for the spectrums of ALD, no satisfactory therapy is available except for abstinence [13,14,15]. Medications that act as anti-inflammatories and anti-oxidants, are frequently used as therapeutic drugs in ALD. For example, there is silymarin, which alleviates liver injury mainly by reducing free radical activity and lipid peroxidation, protecting the liver cell membrane, and promoting hepatocytes regeneration, and bifendate, which alleviates liver injury mainly by reducing serum level of alanine aminotransferase (ALT), inflammatory cell infiltration and liver histological changes. However, clinical applications are limited because of side effects among other reasons. For example, silymarin has poor oral-bioavailability, and bifendate may cause liver hypertrophy [16,17,18]. Thus, finding convincingly effective treatment drugs with fewer side effects without compromising therapeutic effect continues to be an important goal.

Artichoke (*Cynara scolymus* L.), an edible herbal medicine of the family Compositae, is a perennial herb widely studied because of its possible antioxidative and hepatoprotective effects [19,20,21,22]. The extracts and derivatives from artichoke contain a variety of dicaffeoylquinic acids and many kinds of flavonoid functional compounds, such as cynarin (1,5-dicaffeoylquinic acids), chlorogenic acid (3-caffe-oylquinic acid), luteolin glucoside, and apigenin glucoside [23,24], which exhibit anti-microbial, anti-allergic, anti-inflammatory, and anticancer effects. One study indicated that artichoke extract had potential in reducing hypercholesterolemia through preventing lipid peroxidation and ameliorating hepatic antioxidant status [25]. Another report demonstrated that artichoke aqueous leaf extract reduced serum total cholesterol (TC), triglycerides (TG), very low density lipoprotein, glucose levels, and plasma malondialdehyde (MDA) levels in streptozotocin-treated diabetic rats [26]. Reports also demonstrated that artichoke showed marked anti-inflammatory effects on tissue plasminogen activator-induced inflammation and antitumor activity in an in vivo two-stage carcinogenesis test in mice [27]. Besides, it was reported that artichoke extract was very safe to the human body as no obvious side effects were observed after continuous medication for several months [28]. Therefore, artichoke has a broad application prospects in ALD treatment due to its anti-oxidant and anti-inflammatory effects.

The purpose of this study was to investigate the prophylactic protective effects of ethanolic extract from artichoke on acute alcohol-induced injury in an acute ALD mice model. To date, the indicators of aspartate aminotransferase (AST), ALT, TG, TC, MDA, glutathione (GSH), and superoxide dismutase (SOD) were assessed. The possible mechanism for acute alcohol-induced liver injury was discussed using the signals of TLR4 and NF-κB.

## 2. Materials and Methods

### 2.1. Materials

Artichokes in freeze-dried powder (8.89% caffeic acid derivatives, 0.98% chlorogenic acid, 0.56% cynarin) were supplied by Huimei Agricultural Science and Technology Co., Ltd. (Hunan, China), and the artichokes were diluted into 0.04 g/mL, 0.08 g/mL, and 0.16 g/mL suspensions with distilled water, respectively. Edible alcohol was obtained from Beijing Red Star Co., Ltd. (Beijing, China), and edible alcohol was diluted with distilled water to a concentration of 40% (*w*/*v*). Regular chow diet (40–43% corn, 26% bran, 29% bean cake, 1% salt, 1% bone meal, 1% lysine) for mice was purchased from Huayueyang Biotechnology CO., Ltd. (Beijing, China). Bifendate was provided by Beijing Union Pharmaceutical Factory (Beijing, China), and bifendate was diluted as a 0.036 g/mL suspension with distilled water. Diagnostic kits for AST, ALT, TG, TC, SOD, MDA, and GSH were received from the Nanjing Jiancheng Institute of Biotechnology (Nanjing, China). Antibodies for TLR4 and NF-κB p50 were purchased from OriGene Technologies, Inc. (Rockville, MD, USA) and Novus Biologicals, Inc. (Littleton, CO, USA), respectively. All other chemicals used were of analytical reagent and obtained from Sinopharm Chemical Reagent Co., Ltd. (Shanghai, China).

### 2.2. Experimental Animals

Seven-week-old male Institute of Cancer Research (ICR) mice (25 ± 2 g) were supplied by the Shanghai Laboratory Animal Center (Shanghai, China) and acclimated for one week prior to use. These mice were kept under environmentally controlled conditions (12-h normal light/dark cycles, 22 ± 2 °C and 50 ± 10% relative humidity) with chow diet and water ad libitum. All animal experiments were approved by the Animal Care and Use Committee of the Huaqiao University (Approval No. SCXK (HU) 2012-0002) and followed the National Institutes of Health Guidelines for animal care (Approval No. HQ-ECLA-20160517).

The ICR mice were randomly divided into 6 groups of 10 mice per group:

(1) Control group: mice were gavaged with same volume of 0.9% saline twice per day (interval time, one hour).
(2) EtOH group (model group): mice were gavaged with same volume of 0.9% saline and with 12 mL/kg body weight (BW) alcohol one hour after saline administration per day.
(3) Positive control group (EtOH + bifendate): mice were gavaged with 0.36 g/kg BW of bifendate and with 12 mL/kg BW alcohol one hour after bifendate pretreatment each day.
(4) Low-dose artichoke group (EtOH + artichoke 0.4): mice were gavaged with 0.4 g/kg BW of artichoke and with 12 mL/kg BW alcohol one hour after artichoke pretreatment each day.
(5) Middle-dose artichoke group (EtOH + artichoke 0.8): mice were gavaged with 0.8 g/kg BW of artichoke and with 12 mL/kg BW alcohol one hour after artichoke pretreatment each day.
(6) High-dose artichoke group (EtOH + artichoke 1.6): mice were gaveged with 1.6 g/kg BW of artichoke and with 12 mL/kg BW alcohol one hour after artichoke pretreatment each day.

All groups were fed for 10 consecutive days. Then, all groups were fasted for 12 h and subsequently anesthetized by pentobarbital solution (60 mg/kg BW) before the experiment.

### 2.3. Serum Biochemical Assays

Blood samples were collected from the retrobulbar vessels of the mice. The samples were centrifuged at 1500 rpm for 10 min at 4 °C (GTR16-2, Beijing Era Beili Centrifuge Co., Ltd, Beijing, China) to separate the serum after standing for 1 h at room temperature. Serum ALT, AST, TG, and TC activities were subsequently subjected to diagnostic kit testing (Nanjing Jiancheng Institute of Biotechnology) according to the instructions provided using spectrophotometer determination (UV2550, Shimadzu Crop., Kyoto, Japan). Briefly, for assessment of ALT and AST, the samples were mixed with substrates or buffer solution. After incubation at room temperature for 5 min, the absorbance at 505 nm was measured. The final data of ALT and AST were represented as U/L. For assessment of TG and TC, the samples were transferred into a 96-well plate containing substrates or buffer solution. After incubation at 37 °C for 10 min, the plate was incubated for an additional time after adding color developing agent and the absorbance at 510 nm was measured. The final data are represented as μmol/L.

### 2.4. Hepatic Antioxidant and Oxidative Stress Marker Assays

The livers were weighed accurately. A total of 0.5 g liver tissue was cut and washed with distilled water, then excess water was dried up. Then the liver tissue was cut into slices and homogenated with nine volumes of phosphate buffer (4.5 mL) in an ice bath (pH 7.2–7.4). The resulting suspension was centrifuged at 12,000 rpm for 10 min at 4 °C (GTR16-2, Beijing Era Beili Centrifuge Co., Ltd, Beijing, China), and the supernatant was measured by diagnostic kits of SOD, MDA, and GSH (Nanjing Jiancheng Institute of Biotechnology) according to the manufacturer’s instructions. In brief, the concentrations of SOD, MDA and GSH were assayed by hydroxylamine method, thiobarbituric acid-reactive method and microplate method, respectively.

### 2.5. Histological Examination of Liver Tissue

Liver tissues were fixed in 10% neutral formalin buffer for 24 h, and 5-μm sections were cut and stained with hematoxylin and eosin (H&E), and then observed using a Nikon DS-Fi2 fluorescent microscope (Nikon, Tokyo, Japan). The magnification was 200×. At least 10 areas of each tissue slice were observed. Representative images were presented. Analyses of pathological changes were based on proportion of inflammation, necrosis (0 point, 0 foci; 1 point, <2 foci; 2 points, 2–4 foci; 3 points, >4 foci, per 200× field) and steatosis (0 point, <5%; 1 point, 6–33%; 2 points, 34–66%; 3 points, >66%), which were assessed by three examiners independently [29].

### 2.6. Immunohistochemical Analysis of TLR4 and NF-κB

Liver tissues were fixed in 10% neutral formalin buffer and embedded in paraffin. Five-millimeter-thick paraffin sections were cut and were then heated in unmasking solution at 95 °C for 15 min after deparaffinization. Nonspecific binding sites were blocked with goat serum. Sections were then incubated overnight at 4 °C in a humidified chamber with the following primary antibodies: rabbit anti-TLR4 (1:50) and rabbit anti-NF-κB p50 (1:250). The examiners, blinded to the experimental groups, counted the cells labeled with TLR4 and NF-κB p50 throughout five random lesion regions in the stained areas under a 200× light microscope. Then, the expression levels of TLR4 and NF-κB p50 were analyzed by mean integrated optical density (IOD).

### 2.7. Statistical Analysis

All quantifications for assays were repeated for three times and a mean value was used by taking mean of the triplicate plus/minus standard deviation (mean ± SD) for each group. Statistically significant differences (*p* < 0.05) were evaluated by one-way analysis of variance using SPSS 18.0 (SPSS Inc., Chicago, IL, USA).

## 3. Results and Discussion

### 3.1. Liver Index of ICR Mice

The liver index was calculated as a ratio (%) of the liver weight (g) to body weight (g) [30,31]. At the first stage of ALD formation, excessive drinking implies a large number of calories, resulting in a dramatic increase in liver weight, body weight and liver index. Thus, the liver index is an informative ratio for predicting ALD. Table 1 shows the liver indices of mice of six groups. No significant difference was observed in the final body weight in bifendate or artichoke treatment mice compared with the EtOH group. However, the weight gain of mice treated with alcohol decreased significantly (*p* < 0.05) when compared to control group, which could be explained as a symptom of anorexia caused by liver damage, indicating the successful establishment of an acute alcohol-induced mice model. Liver index was significantly increased by 8.79% (*p* < 0.05) in the EtOH group in comparison to the control group, whereas no significant decrease was shown in the bifendate group or artichoke treatment groups compared with the EtOH group. However, the medication groups under bifendate and artichoke treatment all showed a protective effect on the liver as evidenced by comparatively low liver indexes when compared with EtOH group, suggesting that artichoke is helpful for attenuating ethanol-induced liver injury.

### 3.2. Serum Biochemical Markers

The levels of serum ALT, AST, TG and TC are early biochemical and pathological markers of hepatocyte damage. ALT is a cytosolic enzyme which is mainly presented in the cell cytoplasm, while AST is a mitochondrial enzyme which is released from the liver and other organs in the body. ALT and AST are released into the blood, resulting in an increase of serum transaminase when damage (e.g., inflammation and necrosis) occurs in liver cells. Meanwhile, TG and TC are characterized as indicators of fat accumulation in the liver and responses to alcohol consumption [11,32].

**Figure 1:**
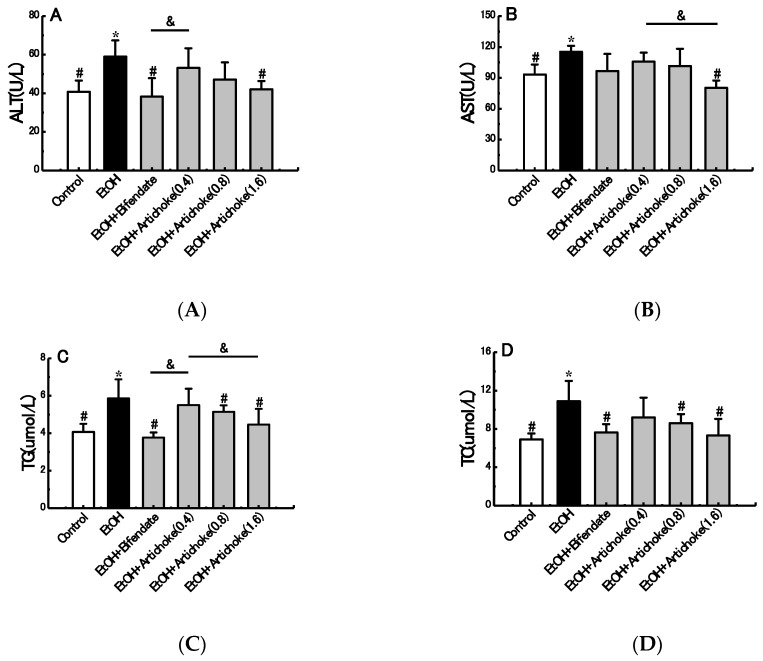
Effects of artichoke on levels of ALT (**A**), AST (**B**), TG (**C**), and TC (**D**). Values represent means ± standard deviation (SD) (*n* = 10); * *p* < 0.05 vs. the control group; ^#^ *p* < 0.05 vs. the EtOH group; ^&^ *p* < 0.05. ALT: alanine aminotransferase; AST: aspartate aminotransferase; TG: triglycerides; TC: total cholesterol.

As shown in Figure 1, serum levels of ALT, AST, TG, and TC in the EtOH group greatly increased by 44.63% (*p* < 0.05), 23.69% (*p* < 0.05), 43.98% (*p* < 0.05), and 57.83% (*p* < 0.05), respectively, compared with the control group. In AST and TG levels, significant decreases were observed between low-dose (0.4 g/kg BW) and high-dose (1.6 g/kg BW) artichoke groups. Notably, the ALT, AST, TG, and TC levels in the artichoke pretreatment groups were abated in a dose-dependent manner compared with the EtOH group. Pretreatment with high-dose artichoke (1.6 g/kg BW) markedly recovered serum ALT, AST, TG, and TC levels to near those of control group mice. The results demonstrated that artichoke pretreatment was also effective in reversing the acute alcohol-induced liver dysfunction.

### 3.3. Histopathological Analysis

**Figure 2:**
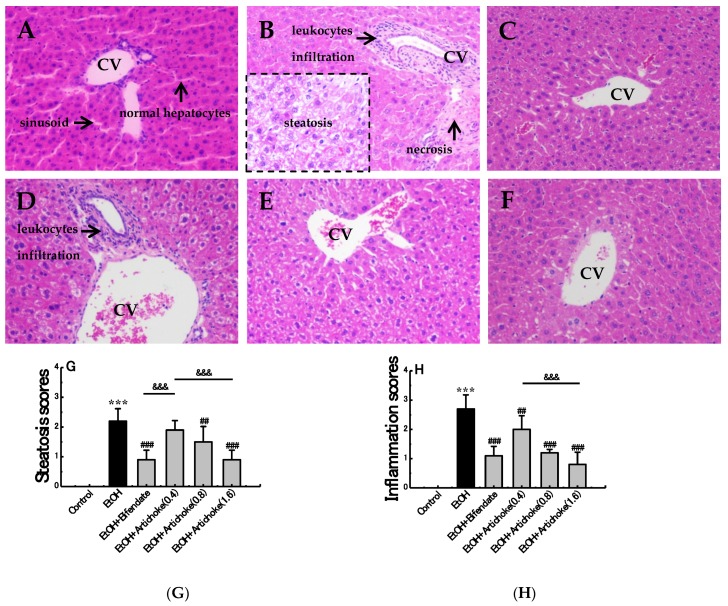

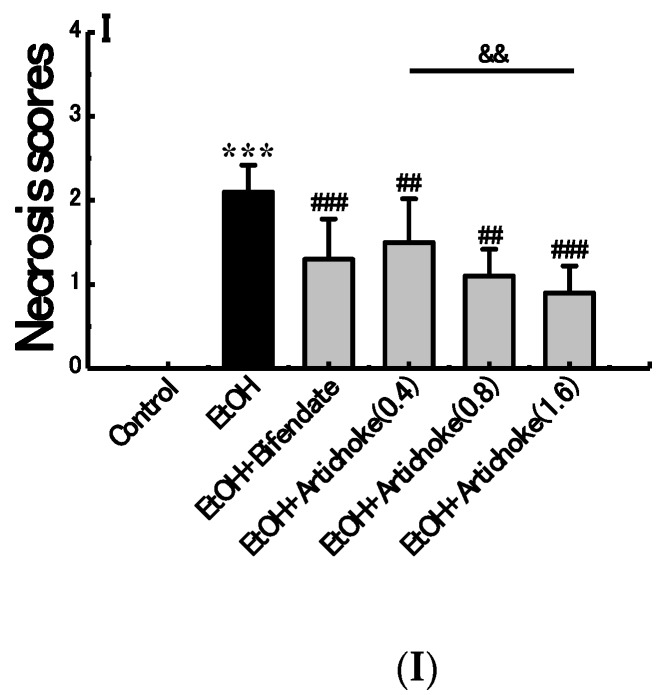
Effect of artichoke on alcohol-induced histopathological changes in liver tissues. (**A**) Control group, (**B**) EtOH group, (**C**) positive control group, (**D**) low-dose group, (**E**) middle-dose group, (**F**) high-dose group, (**G**) steatosis scores, (**H**) inflammation scores, (**I**) necrosis scores, and central vein (CV). Hematoxylin and eosin staining. Original magnification, 200×. Values represent means ± SD (*n* = 10); *** *p* < 0.001 vs. the control group; ^##^ *p* < 0.01, ^###^ *p* < 0.001 vs. the EtOH group; ^&&^ *p* < 0.01, ^&&&^ *p* < 0.001.

The effect of artichoke on the histopathology of the acute ALD mice is presented in Figure 2. Staining with H&E revealed normal hepatic architecture within the complete structure, similar size, tight arrangement, regular hepatic cords with central vein, and clear hepatic sinusoid in control group (Figure 2A). Nevertheless, the EtOH group demonstrated evident pathological changes, including loose arrangement, disarrangement of cell cords, leukocytes infiltration, necrosis, and hepatocytes steatosis, which confirmed the establishment of liver injury with inflammation (Figure 2B). Slight leukocyte infiltration and hepatocyte steatosis was still observed in the low-dose artichoke (0.4 g/kg) group (Figure 2D). However, pretreatment with bifendate and artichokes exerted a regenerative effect of hepatocytes and a decrease of necrotic and inflamed areas. This indicated that artichoke pretreatment promoted structure restoration of the liver to a certain extent.

As shown in score results (Figure 2G–I), the pathological scores of steatosis, inflammation and necrosis increased significantly (*p* < 0.001) in the EtOH group when compared to control group. However, compared with EtOH group, the degree of hepatic change had made an improvement in the artichoke and bifendate pretreatment group, suggesting that artichoke had preventive effect of alleviating alcohol-induced liver steatosis, inflammation and necrosis. Significant differences were observed between artichoke low-dose (0.4 g/kg BW) and high-dose (0.8 g/kg BW) groups in all score results. Interestingly, the decrease effect of artichoke was in a dose-dependent manner.

### 3.4. Hepatic Antioxidant and Oxidative Stress Markers

Oxidative stress plays a central role in alcohol-induced liver injury and ALD pathogenesis [33]. To counterbalance oxidative stress, a number of enzymatic (e.g., SOD) and non-enzymatic (e.g., GSH) mechanisms have evolved to protect against reactive oxygen species (ROS) caused by oxidative stress in alcoholic liver injury. SOD reduces the generation of free radical and lipid peroxide and even accelerates its clearance, thus reducing the damage of liver cells [34,35]. GSH serves as a reservoir for cysteine to counteract ROS [36]. SOD and GSH activities reflect the body’s ability of clearing oxygen free radicals indirectly. Meanwhile, MDA is the main product of lipid peroxidation induced by ROS; in addition, MDA content can reflex the severity of free radical attacks on the body cells indirectly [37,38]. Regarding oxidative stress, excessive ROS and MDA will consume a large quantity of antioxidation factors, such as SOD and GSH. The in vivo SOD and GSH would be unable to fight against the excessively increasing ROS and MDA once the balance is lost. Maintaining suitable levels of SOD, GSH, and MDA plays an important role in liver protection from attacks of free radicals.

**Figure 3:**
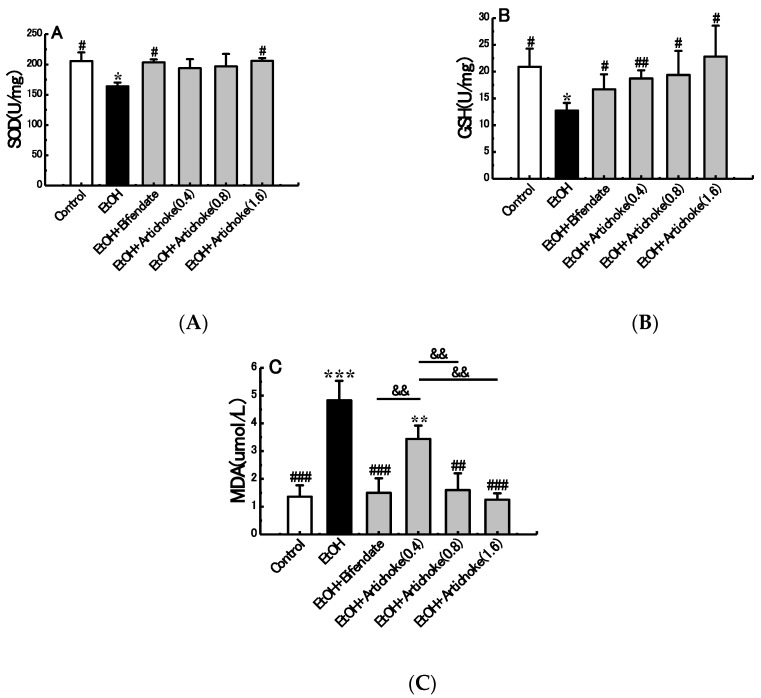
Effects of artichoke on levels of SOD (**A**), GSH (**B**), and MDA (**C**). Values represent means ± SD (*n* = 10); * *p* < 0.05, ** *p* < 0.01, *** *p* < 0.001 vs. the control group; ^#^ *p* < 0.05, ^##^ *p* < 0.01, ^###^ *p* < 0.001 vs. the EtOH group; ^&&^ *p* < 0.01. SOD: superoxide dismutase; GSH: glutathione; MDA: malondialdehyde.

As shown in Figure 3, the SOD and GSH in the EtOH group decreased by 20.24% (*p* < 0.05) and 39.08% (*p* < 0.05) respectively compared with the control group. By contrast, hepatic MDA activity increased significantly by 255.14% (*p* < 0.001) in the EtOH group compared with the control group. Results showed that the activities of SOD and GSH in the blood increased significantly, whereas MDA decreased significantly when artichoke was subjected to an alcohol diet. Notably, a dose-dependent mechanism was found for all SOD, GSH and MDA activities. High-dose artichoke (1.6 g/kg BW) administrations significantly protected against the acute alcohol-induced elevation of MDA activity (*p* < 0.001) and reduction of SOD (*p* < 0.05) and GSH (*p* < 0.05). This suggested that pretreatment of artichoke could protect the liver from acute alcohol-induced hepatic oxidative stress.

### 3.5. TLR4 and NF-κB Expression Levels

TLRs are a family of pattern-recognition receptors that enable the innate immune system to react immediately to infections by recognizing both the bacterial and viral constituents [39]. Among these, TLR4 is responsible for LPS-induced inflammatory reaction. Acute excessive alcohol exposure increases gut permeability, which allows gut-derived endotoxins (LPS) to bind with TLR4. The interaction of TLR4 and LPS result in the activation of NF-κB, which is composed of the p50 and p65 subunits. Generally, NF-κB is sequestered as an inactive complex in cytoplasm by inhibitory subunit-inhibitory κB (IκB). Once activated, IκB is phosphorylated and degraded, which allows NF-κB p50 to translocate to the nucleus and induce the expression of its target genes, hence resulting in release of pro-inflammatory cytokines, such as tumor necrosis factor α (TNF-α), the interleukin (IL)-1 receptor (IL-1β), IL-6, and IL-8. All of these pro-inflammatory mediators can aggravate inflammatory progress, ultimately leading to hepatic fibrosis, cirrhosis, and hepatocellular carcinoma [8,40,41]. Thus, the expression levels of upstream index TLR4 and downstream index NF-κB in an inflammatory pathway are two pivotal indicators implicated in inflammation.

**Figure 4:**
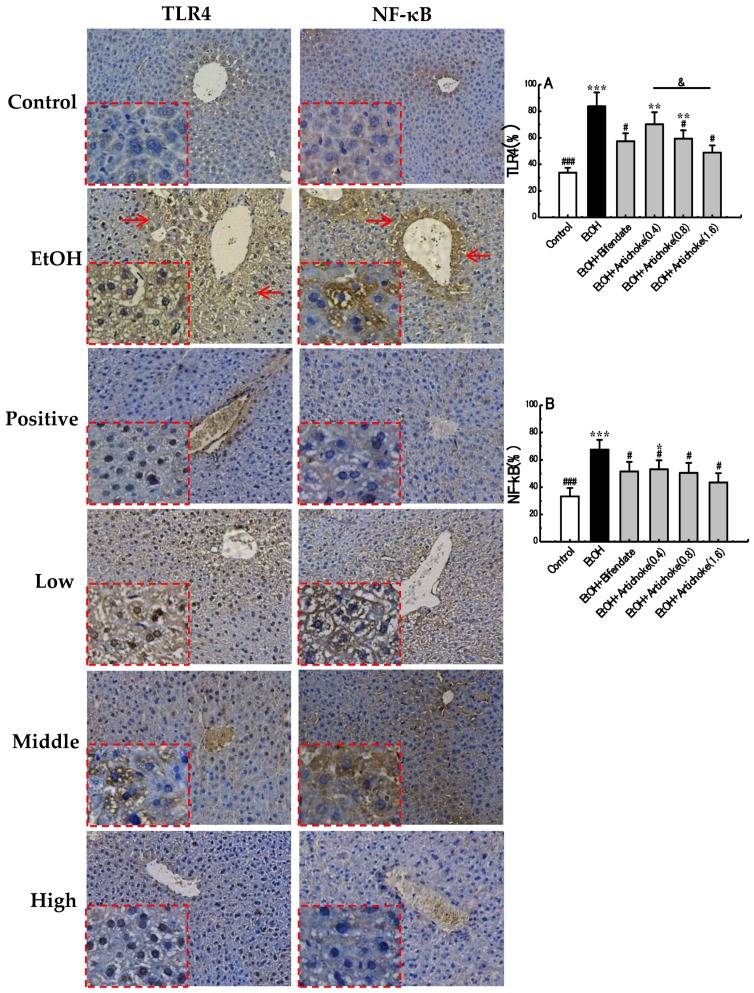
Immunohistochemical analysed results of mice liver in TLR4 and NF-κB. (**A**) mean IOD of TLR4, (**B**) mean IOD of NF-κB, and (**Red Arrows**) positive cells. Original magnification, 200×. * *p* < 0.05, ** *p* < 0.01, *** *p* < 0.001 vs. the control group; ^#^ *p* < 0.05, ^###^ *p* < 0.001 vs. the EtOH group; ^&^ *p* < 0.05. TLR: toll-like receptor; NF-κB: nuclear factor-kappa B; IOD: integrated optical density.

As shown in Figure 4, the positive cells of TLR4 were stained as brown or yellow, which were distributed mainly in the cytoplasm and cell membrane, whereas the positive cells of NF-κB p50 were stained as brown in the nucleus. The mean IOD result showed that expression levels of TLR4 and NF-κB p50 in the EtOH group were elevated significantly, by 147.88% (*p* < 0.001) and 103.58% (*p* < 0.001), respectively, compared with the control group. However, these increases were attenuated by artichoke pretreatment in a dose-dependent manner compared with the EtOH group. In the mean IOD of TLR4 expression, a significant difference (*p* < 0.05) was observed between low-dose (0.4 g/kg BW) and high-dose (1.6 g/kg BW) artichoke pretreatment groups. Based on the results, the preventive effect of artichoke against acute alcohol-induced liver injury was associated with TLR4 downregulation. The activation of NF-κB was therefore inhibited. Thus, the release of pro-inflammatory cytokines was limited. The inflammatory effect was then ultimately suppressed.

## 4. Conclusions

Data presented clearly showed that artichoke reduced levels of AST, ALT, TG, TC, and MDA, while increasing the levels of SOD and GSH in an acute ALD mice model. Degeneration and necrosis of hepatic cells were also significantly attenuated by artichoke. Besides, TLR4 and NF-κB expression levels in liver tissue were effectively downregulated by artichoke. Our study demonstrated that 1.6 g/kg BW artichoke exhibited significant preventative potentiality for acute alcohol-induced liver injury, whereas the effect of 0.4 g/kg BW artichoke was not significant. The preventative effect of artichoke was mainly due to its ability to attenuate oxidative stress and inhibit the inflammatory pathway by suppressing the expression levels of TLR4 and NF-κB. Overall, 1.6 g/kg BW artichoke could be an effective adjuvant in the prevention of acute ALD. Moreover, interventions in the pathogenesis of the TLR4/NF-κB pathway showed its potential as a therapeutic target in acute alcohol-induced liver injury.

## Figures and Tables

**Table 1:** Effect of artichoke on liver index of acute alcoholic liver disease (ALD) mice ^a^.

Treatment Group	Dosage (g/kg)	Liver Weight (g)	Initial Body Weight (g)	Final Body Weight (g)	Liver Index (%)
Control	_	1.75 ± 0.22	26.46 ± 0.64	36.96 ± 4.26 ^#^	4.78 ± 0.64 ^#^
EtOH	_	1.53 ± 0.17	26.24 ± 0.70	29.94 ± 2.98 *	5.20 ± 0.22 *
EtOH + Bifendate	0.36	1.53 ± 0.18	26.17 ± 0.95	30.18 ± 2.13 *	5.05 ± 0.38
Low-dose artichoke	0.4	1.47 ± 0.23	26.35 ± 0.72	28.79 ± 3.00 *	5.12 ± 0.64
Middle-dose artichoke	0.8	1.57 ± 0.20	26.39 ± 0.52	29.06 ± 2.03 *	5.04 ± 0.44
High-dose artichoke	1.6	1.54 ± 0.12	25.94 ± 0.85	29.84 ± 1.27 *	5.14 ± 0.31

^a^ Data are expressed as means ± standard deviation (SD) (*n* = 10); * *p* < 0.05, vs. the control group; ^#^ *p* < 0.05, vs. the EtOH group.
